# Malware detection using pre-trained transformer encoder with byte sequences

**DOI:** 10.1371/journal.pone.0332307

**Published:** 2025-10-13

**Authors:** Eun-Jin Kim, Yun-Kyung Lee, Sang-Min Lee, Jeong-Nyeo Kim, Ah Reum Kang, Mi-seo Kim, Young-Seob Jeong

**Affiliations:** 1 Department of Computer Engineering, Chungbuk National University, Cheongju, Chungcheongbukdo, Republic of Korea; 2 Cyber Security Research Division, Electronics and Telecommunications Research Institute, Yuseong-gu, Daejeon, Republic of Korea; 3 Department of Information Security, Pai Chai University, Daejeon, Republic of Korea; Lucian Blaga University of Sibiu: Universitatea Lucian Blaga din Sibiu, ROMANIA

## Abstract

Ordinary users encounter various documents on the network every day, such as news articles, emails, and messages, and most are vulnerable to malicious attacks. Malicious attack methods continue to evolve, making neural network-based malware detection increasingly appealing to both academia and industry. Recent studies have leveraged byte sequences within files to detect malicious activities, primarily using convolutional neural networks to capture local patterns in the byte sequences. Meanwhile, in natural language processing, Transformer-based language models have demonstrated superior performance across various tasks and have been applied to other domains, such as image analysis and speech recognition. In this paper, we introduce a novel Transformer-based language model for malware detection that processes byte sequences as input. We propose two new pre-training strategies: real-or-fake prediction and same-sequence prediction. Including conventional pre-training strategies such as masked language modeling and next-sentence prediction, we explore all possible combinations of these approaches. By compiling existing byte sequences for malware detection, we construct a benchmark consisting of three file types (PDF, HWP, and MS Office) for pre-training and fine-tuning. Our empirical results demonstrate that our language model outperforms convolutional neural networks in the malware detection task, achieving a macro F1 score improvement of approximately 2.7%p∼11.1%p. We believe our language model will serve as a foundation model for malware detection services, and will extend our research to develop a more powerful encoder-based model that can process longer byte sequences.

## Introduction

Online users are vulnerable to malicious programs or files, especially non-executables such as Microsoft Excel, Word, PowerPoint, and Hangul documents, where Hangul is a widely-used document software in South Korea. When people find such non-executable attachments in their mailbox, they are often not very cautious when opening the documents. This may harm their systems or cause severe damage to important files (e.g., customer databases), so it is necessary to develop a method that analyzes and detects malicious non-executables. There are mainly two ways to perform malware analysis: static and dynamic. The dynamic method involves a separate platform or an isolated virtual environment and examines step-by-step actions of a suspicious program. Existing studies of this method have a limitation in that they are not reproducible on different emulations. On the other hand, the static method analyzes the suspicious file without running it, making it preferable as the number of files on online platforms continues to increase.

**Fig 1:**
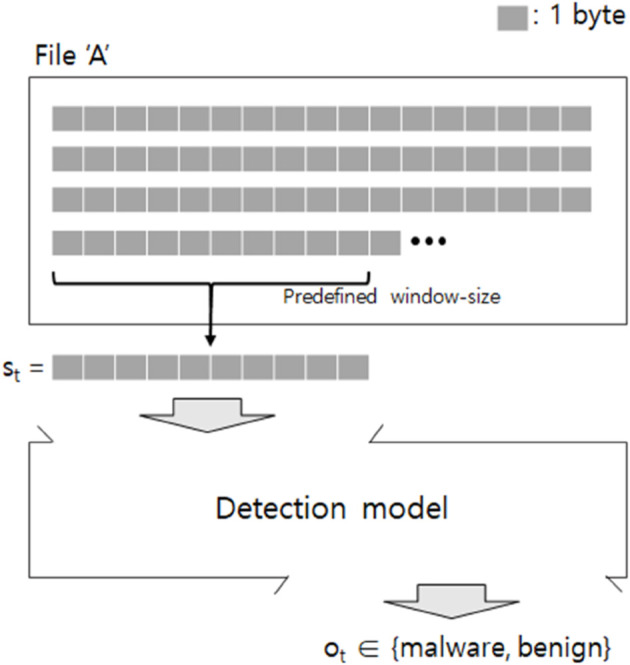
Overview of malware detection using byte sequences.

Recently, studies have exploited byte sequences within non-executables for the task of malware detection. These studies assume that there are sequential patterns underlying the byte sequences within the files, since the files contain semantic content following particular formats (e.g., portable document format, docx). The general process of them is depicted in Fig 1. Suppose that the file ‘A’ is a non-executable containing multiple byte sequences, and the set of sub-sequence samples $S=\{s_{1},...,s_{\left|S\right|}\}$ can be obtained in a sliding-window manner. The detection model at the bottom of the Fig 1 takes the *t*-th sample *s*_*t*_ as input where 1≤t≤|S|, and gives the prediction output *o*_*t*_. When we have *o*_*t*_ = malware for any *t*, the file ‘A’ is considered suspicious, and we can take some action (e.g., deletion) on the file. This paper proposes a new detection model that is pre-trained with a large amount of byte sequences, and we demonstrate its superior performance compared to previous detection models.

Although previous studies on malware detection using byte sequences have shown promising results, this line of work has common issues. First, only a few datasets are publicly available (e.g., MS Office dataset [1]). One may argue that we may borrow public datasets (e.g., Virusshare). However, not all document-type files can be used for training because the objects within the file must have labels indicating which parts correspond to malicious actions. Annotating these labels requires expert input and is costly, making publicly available annotated datasets scarce. The lack of datasets makes it difficult to reproduce and compare existing methods. Second, there are only a few Transformer-based studies. Since the Transformer was introduced [2] in the field of natural language processing (NLP), many variants and language models have emerged, such as Bidirectional Encoder Representations from Transformers (BERT) [3] the Generative Pre-trained Transformer (GPT) series [4–6]. Although Transformer-based methods have shown successful performance in many tasks not only in the NLP field but also in image analysis, speech analysis, and other areas, there have been only a few Transformer-based studies for malware detection using byte sequences.

Some previous studies have adopted Graph Neural Networks (GNNs) [7,8], or combined Convolutional Neural Networks (CNNs) with Recurrent Neural Networks (RNNs) [9], to tackle different problems, and the CNNs have often been adopted as a malware detection model because CNN-based models are known to be effective in capturing local patterns [1,10,11]. These studies assumed that there exist useful local patterns in the byte sequences, and the convolution operations were used to capture the patterns. Even though the byte sequences are often compressed or encrypted, the studies revealed that the CNN-based models effectively work by capturing the local patterns in the byte sequences. For long byte sequences, they proposed a shallow-wide architecture or a stack of convolutions.

Since Transformer [2] has appeared, its variants achieved success in not only the natural language processing (NLP) area, but also in malware detection and classification. The most well-known Transformer variants are BERT and GPT series, where the BERT and GPT are the encoder and decoder part of the Transformer, respectively. There are few studies that employed the GPT (transformer decoder) for malware detection tasks. Nazenın şahın [12] proposed a method that feeds the assembly code obtained from a static analysis on PE files to GPT-2 language model [5]. In Denız Demırcı et al. [13], the assembly instructions are used as an input for a stacked long-short term memory (LSTM) [14] and GPT-2 language model. While the above studies adopted the Transformer decoder, more studies have employed BERT (Transformer encoder) since BERT is known to be more efficient and powerful in natural language understanding (NLU) tasks, whereas the GPT series is effective in natural language generation (NLG) tasks. The malware detection and classification tasks are NLU-related tasks, making the BERT-based approach preferable. For example, MalBERT is a BERT-based approach that takes source codes of Android applications as input and generates distributed representations used for malware classification [15]. They proved that MalBERT was superior to LSTMs through experimental results. In Ferhat Demirkıran et al. [16], a pre-trained CANINE BERT [17] was borrowed for API call sequence analysis. They showed that their method achieves state-of-the-art performance in malware classification. MalBERTv2 [18] was an extension of their prior work (i.e., MalBERT), incorporating new pre-tokenization and a complete pipeline for malware analysis. All previous studies using Transformer architecture have indicated that pre-training with a large amount of data allows the model to learn arbitrary knowledge, so that the model achieves higher performance in target tasks by better understanding the underlying patterns of the tasks.

In this paper, we treat the byte sequences within non-executables as character sequences within documents, and find an answer to a question: “What if a Transformer-based model is pre-trained with a large amount of byte sequences? Will it work for malware detection task?” We designed and constructed a new Transformer encoder-based pre-trained model that learns the representation of byte sequences within files, namely ByteEnc. As far as we know, this is the first study to pre-train an encoder-based language model using only byte sequences. Furthermore, we design new pre-training strategies and investigate their impact through experimental results on the malware detection task. Our contributions can be summarized as follows:

- **Pre-training algorithm**: We design new pre-training algorithms and examine possible combinations with other pre-training algorithms to improve task performance.
- **Pre-trained model**: We introduce our Transformer encoder-based pre-trained model, namely ByteEnc. As far as we know, this is the first encoder-based model that is pre-trained exclusively with byte sequences of non-executable documents. We demonstrate the effectiveness of the pre-trained model by performing experiments on three file formats (e.g., Portable Document Format (PDF), Hangul Word Processor (HWP), and Microsoft (MS) Office).

## Materials and methods

### Materials

**Fig 2:**
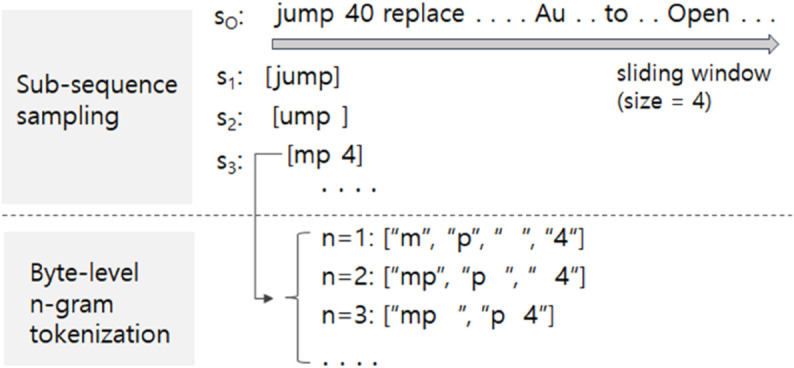
(Top) An example of sub-sequence sampling from a raw byte sequence using a sliding-window technique with a window size of 4, and (bottom) an example of byte-level n-gram tokenization for the given sub-sequence $s_{3}$.

Although there have been studies on malware detection using byte sequences, most of them did not disclose their datasets, and only a few public datasets are available, as follows. In Young-Seob Jeong et al. [11], byte sequences of PDF documents were provided as files of comma-separated values (CSV) format. This dataset is downloadable from a public website (https://sites.google.com/view/datasets-for-public/). A new CNN-based design was proposed for malware detection in Hangul Word Processor (HWP) documents as a defensive measure to protect governmental institutes of South Korea from North Korea, and their HWP dataset is available upon request [19]. Byte sequences of Microsoft (MS) Office documents (e.g., MS Word, MS PowerPoint) were provided [1]. We utilize all available datasets above and make them into a uniform format for convenient use. Following previous studies [1,10,20], we extract sample sequences (i.e., sub-sequences) of a predetermined length, as the existing datasets contain byte sequences of varying lengths. Specifically, considering the byte sequence *s*_*O*_ in Fig 2, the sub-sequences were randomly extracted, with a maximum of 100 sub-sequences from the same byte sequence. The extracted samples are 512 bytes in length.

From the available byte-sequence datasets above, we constructed two different datasets for experiments: (1) a task dataset for malware detection and (2) a pre-training dataset. The pre-training dataset was used only for the pre-training phase, and the task dataset was used to finetune the pre-trained LM for malware detection. A summary of the datasets is described in Table 1. The raw file contains one or more byte sequences, and each byte sequence is annotated with l∈{malware, normal}. Since the raw file provides an exact offset (i.e., position) of a malicious action, the malware sub-sequences are sampled to ensure they contain the offset. For convenience, we rearrange the raw files of different formats to have the same template. Furthermore, we also provide the sampled sub-sequences in a format readable by the Transformers library. We checked the licenses of all data sources and confirmed that it is allowed to disclose our reformed dataset.

**Table 1:** Summary of datasets where (n:m) represents ‘normal’ and ‘malware’, respectively.

	Format	Raw files(n:m)	Samples		Data source
			train(n:m)	test(n:m)	
Task	PDF	2,507:8,223	24,061:8,324	2,378:969	Public website [11]
	HWP	223:98	79,327:29,761	7,433:1,831	Authors [19]
	MSOffice	551:511	45,305:14,385	6,428:1,825	Public website [1]
Pre-training	HWP	1,003:510	825,918	-	Authors [19]

### ByteEnc

The architecture of our proposed language model, ByteEnc, is the same as that of BERT; ByteEnc has stacked Transformer encoders. However, it is not trivial to make ByteEnc work properly with byte streams because of two issues: tokenization and positional encoding.

#### Tokenization.

The first issue is tokenization, which arises from the different nature of byte sequences compared to natural language texts. The Transformer and its variants were originally designed for natural language processing, but byte sequences differ significantly from natural language sentences. For example, byte sequences are often much longer than sentences; as reported in Young-Seob Jeong et al. [21], HWP byte sequences range from 350K to 710K bytes. It is not trivial to take such extremely long sequences as input, so many previous studies sliced the original sequences into smaller segments (i.e., sub-sequences) using a sliding-window technique.

The previous studies employing the sub-sequence sampling applied simple byte-level tokenization followed by an embedding layer that maps each byte into an embedding space. The reason for utilizing byte-level tokenization is that operational boundary-based tokenization is vulnerable to the out-of-vocabulary (OOV) problem. That is, when constructing a vocabulary consisting of operational keywords (e.g., goto, replace, ...), new keywords may appear in byte sequences due to program version updates or the emergence of new file types. Such OOV keywords, which are not present in the vocabulary, can significantly degrade the generalizability of models. Another reason is that the sub-sequences are not consistent with operation boundaries. Consider the two sub-sequences *s*_2_ and *s*_3_ at the top of Fig 2. They begin in the middle of an operation (e.g., jump), and such inconsistency with the operation boundary may cause poor tokenization if NLP tokenizers (e.g., byte-pair encoding (BPE) tokenizer) are used. The byte-level tokenization alleviates this problem by simply converting a sub-sequence into a sequence of bytes, defining each byte as a token. However, the byte-level tokenization may lose arbitrary semantic patterns (e.g., operation keywords) within the sub-sequences. To systematically examine this, we formulate the previous byte-level tokenization as a special type (i.e., *n*=1) of byte-level n-gram tokenization. The bottom of Fig 2 describes how the byte-level n-grams work. For example, the sub-sequence *s*_3_ will be converted into a sequence of four tokens when *n*=1, three tokens when *n*=2, and two tokens when *n*=3. Empirical results with different settings of *n* in the Results and discussion section will show that the best performance is achieved when *n*=1.

#### Positional encoding.

The second issue is positional encoding. Language models in the NLP field, such as BERT variants and the GPT series, rely on specific positional encoding. There are mainly two types of positional encoding algorithms: absolute and relative. The most well-known absolute positional encoding is the Sinusoid positional encoding of the Transformer [2], while a few widely used algorithms utilize relative positional information: relative positional embedding [22], AliBi [23], and rotary positional embedding [24]. Applying absolute positional encodings to byte sequences is not appropriate because byte sequences differ from natural language texts. As stated in Young-Seob Jeong et al. [19], a malicious action within a byte sequence may consist of small fractions scattered throughout the sequence. Suppose a set *S* of sub-sequences is sampled from a long byte sequence with randomly scattered fractions of a malicious action. In that case, absolute positional information within every sub-sequence $s_{i}\in S$ will not be informative. Therefore, instead of absolute positional encoding, we use relative positional encoding; specifically, we adopt the relative positional embedding (RePE) [22] in this paper.

One may argue that the RePE is not useful because malicious actions may appear in random positions within each sub-sequence. To investigate this, we also try not using any positional encodings, which is equivalent to the no-positional-encoding (NoPE) [25]. Experimental results will show that the RePE gives better performance than the NoPE.

### Pre-training strategy

There have been several pre-training (PT) algorithms developed so far. Two representative PT algorithms for Transformer encoder-based models are masked-language modeling (MLM) and next-sentence prediction (NSP) [3]. We adopt these algorithms but also propose two new algorithms: Real-or-Fake Prediction (RFP) and Same-Sequence Prediction (SSP), which are designed according to the distinct characteristics of byte sequences.

#### Masked-language modeling.

The most well-known PT algorithm is masked-language modeling (MLM), which is used for pre-training BERT models [3]. The MLM algorithm introduces noise to the input sequence by replacing some original tokens with ‘mask’ tokens or random tokens. This enables the language model (LM) to comprehend the context of the given sequence, which, in turn, enhances its performance on natural language understanding (NLU) tasks. We adopt the MLM algorithm with a masking probability of *p*_*mlm*_ = 0.15. For more details of the MLM algorithm, please refer to Jacob Devlin et al. [3].

#### Real-or-fake prediction.

In Kevin Clark et al. [26], the replaced token detection (RTD) algorithm was proposed to train the discriminator component of ELECTRA. The discriminator takes a token sequence, part of which is corrupted (i.e., replaced by tokens sampled from the generator), and is tasked with predicting which tokens match the original, uncorrupted sequence. Motivated by the RTD algorithm, we design a new algorithm called Real-or-Fake Prediction (RFP), which can be seen as a special type of token corruption prediction (TCP) [27].

**Fig 3:**
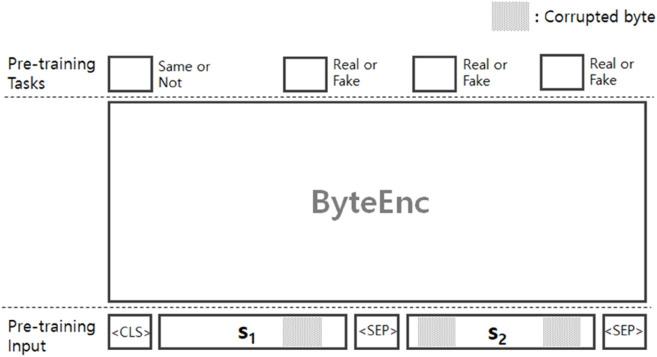
Overview of the new pre-training algorithms: Real-or-Fake Prediction (RFP) and Same-Sequence Prediction (SSP).

The RFP algorithm introduces noise to part of the input sequence by replacing the original tokens with randomly chosen fake tokens. The algorithm has two probabilities: *p*_*rfp*1_ and *p*_*rfp*2_. It first selects candidate tokens with the probability *p*_*rfp*1_, and each candidate token is replaced by a random (fake) token with the probability *p*_*rfp*2_; thus, half of the candidate tokens will be fake, and the other half will be true tokens. This requires the language model (LM) to predict whether each token within the sequence is ‘real’ (uncorrupted) or ‘fake’ (corrupted), essentially making it a token-level binary classification task. As depicted in Fig 3, ByteEnc takes the two sequences *s*_1_ and *s*_2_ as input, where <CLS> and <SEP> are a classification token and a separation token, respectively. A part of the two sequences is selected as candidate bytes, and the ByteEnc predicts whether these bytes are fake or not.

The biggest difference between the RFP and MLM algorithms is that RFP is a token-level binary classification, whereas MLM is a token-level multi-class classification. Since byte sequences may contain randomly scattered fractions of malicious actions and are often compressed or encrypted, we believe that the MLM algorithm is too challenging, as it requires the LM to predict the correct bytes among compressed, encrypted, and randomly scattered bytes. In contrast, the RFP algorithm is easier than the MLM algorithm because it is a binary classification task, and we assume it provides a more suitable level of difficulty for the pre-training objective.

#### Next-sentence prediction.

The next-sentence prediction (NSP) algorithm is a sequence-level binary classification; given a sequence pair (*s*_*i*_, *s*_*j*_), it predicts whether *s*_*j*_ is the next sequence following *s*_*i*_. With a probability *p*_*nsp*_ = 0.5, *s*_*j*_ will be the next sequence, i.e., *j* = *i*  +  1; otherwise, *j* will be a random index. The NSP algorithm was used to pre-train BERT, and we adopted the NSP algorithm to examine its combination with the MLM or RFP algorithms. For more details of the NSP algorithm, please refer to Jacob Devlin et al. [3].

#### Same-sequence prediction.

Some previous studies have reported that the NSP algorithm might not be beneficial for downstream task performance because it is semantically shallow and can often be solved easily through lexical overlap [27,28]. We also believe that the NSP algorithm is too simplistic for byte sequences, so we propose an alternative algorithm, namely Same-Sequence Prediction (SSP). Given a sequence pair (*s*_*i*_, *s*_*j*_), the SSP algorithm assumes that *s*_*i*_ and *s*_*j*_ are sub-sequences sampled from an original sequence. With a probability *p*_*ssp*_ = 0.5, *s*_*j*_ is sampled from the same original sequence as *s*_*i*_; otherwise, *s*_*j*_ is sampled from a different sequence. This approach requires the language model (LM) to predict whether the two sub-sequences originate from the same original sequence, making it a sequence-level binary classification task. Specifically, as shown in Fig 3, the output representation of the <CLS> token is used to generate the sequence-level prediction. Even if the sub-sequences are from the same sequence, they might be quite far apart, making this task more challenging than the NSP algorithm. We believe that the SSP algorithm encourages the LM to understand patterns at the original sequence level, which may improve task performance.

#### Combinations.

Based on the finding that a combination between the MLM with other PT algorithms contributed to task performance improvement [27], we investigate combinations between the PT algorithms. The above-mentioned four PT algorithms can be divided into two groups: token-level and sequence-level. The MLM and RFP algorithms are token-level, while the remaining two algorithms are sequence-level. We examine four combinations: MLM+NSP, MLM+SSP, RFP+NSP, and RFP+SSP. Fig 3 depicts an overview of the combination of our two newly proposed algorithms: Real-or-Fake Prediction (RFP) and Same-sequence prediction (SSP). Algorithm 1 describes step-by-step process of data preparation for the combination of RFP and SSP algorithms. The ByteEnc takes a concatenation or a pair of two byte sequences *s*_*i*_ and *s*_*j*_ (see the line 2 in Algorithm 1). From a resulting vector of the first byte (i.e., <CLS>), we get a result of binary classification according to the SSP algorithm. The line 3 in Algorithm 1 generates a label for the binary classification. At the same time, the sequence pair may contain noise bytes (fake bytes) where these bytes are selected using the probability *p*_*rfp*_ (see line 4 ∼ 10). For each noise byte we get its resulting vector through a forward pass, and make a byte prediction according to the RFP algorithm.

**Algorithm 1 Data preparation for RFP+SSP combination.**

**Require:** input sequence $S=\{s_{1},s_{2},...\}$, two probabilities of RFP

algroithm *p*_*rfp*1_ and *p*_*rfp*2_

1: randomly pick i and j from {1,2,...,|S|}

2: a pair of two sequences $X=[<CLS>,s_{i},<SEP>,s_{j},<SEP>]$

3: *y*_*SSP*_ = 1 if *s*_*i*_ and *s*_*j*_ came from the same original stream,

otherwise 0

4: *x*_*RFP*_ = *[*0,0,...,0*]* where |*x*_*RFP*_| = |*X*|

5: *y*_*RFP*_ = *X*

6: **for** *k* = 0 to |X| **do**

7:   **if** *X*_*k*_ not in {<CLS>,<SEP>} and g~Bernoulli(*p*_*rfp*_)=1  **then**

8:    *y*_*RFP*,*k*_ = randomly chosen byte with a probability *p*_*rfp*2_

9:   **end if**

10: **end for**

## Results and discussion

### Settings

We try many settings including different positional embeddings, n-grams, and combinations of pre-training strategies. Specifically, NoPE and RePE are considered for the positional embeddings, while unigram and bigram are evaluated for n-grams. For the combinations of pre-training algorithms, we try four possible combinations (e.g., MLM+NSP, MLM+SSP, RFP+NSP, and RFP+SSP) and every single algorithm (e.g., RFP, NSP, SSP, and MLM). As it is not trivial to pre-train big language models for all different settings, we chose a small language model, BERT-small, having 4 encoder layers with hidden dimension 512, and 8 attention heads. To check scalability, we also try a greater model, BERT-base, and its results are described at the end of this section.

We employed the AdamW optimizer [29] and the cross entropy loss for both pre-training and fine-tuning. The hyper parameters for pre-training are as follows: initial learning rate *lr* = 5*e* − 05, $\beta_{1}=0.9$, $\beta_{2}=0.999$, ϵ=1e − 08, a weight decay *wd* = 0.001, mini-batch size *bs* = 128, and warmup steps *ws* = 1000. The number of epochs was 100 and 10 for the pre-training and fine-tuning, respectively. The performance metrics are precision, recall, and F1 score. For all experimental results, we conducted three independent runs and computed average performance. The specification of our machine is summarized in Table 2.

**Table 2:** Machine specification.

Item	Specification
CPU	AMD Ryzen Threadripper PRO 5955WX 16-Cores
RAM	512GB
GPU	NVIDIA GeForce RTX 4090 (2EA)

### Positional encoding and n-grams

Before we compare different pre-training algorithms for malware detection, we firstly conducted experiments to find the best settings of positional encoding and n-grams. As described in the Materials and method section, we compare the NoPE and RePE by the malware detection performance and also try n∈{1,2} for the byte-level n-gram tokenization. The averaged results are summarized in Table 3.

**Table 3:** Averaged malware detection performance of the ByteEnc (BERT-small) pre-trained with the MLM algorithm, where NoPE and RePE indicate the relative positional embedding and No positional encoding, respectively.

Positional encoding (n-grams)	Format	Normal			Malware		
		Precision	Recall	F1	Precision	Recall	F1
NoPE (n=1)	PDF	99.26	99.59	99.42	98.99	98.18	98.59
	HWP	82.89	97.95	89.79	68.43	17.93	28.41
	MSOffice	91.91	94.26	93.07	77.80	70.77	74.12
NoPE (n=2)	PDF	99.01	99.55	99.28	98.88	97.53	98.20
	HWP	83.46	98.91	90.53	81.28	19.51	31.46
	MSOffice	92.76	93.92	93.34	68.72	64.49	66.51
RePE (n=1)	PDF	99.44	99.52	99.48	98.83	98.63	98.73
	HWP	83.19	98.71	90.29	78.43	19.06	30.67
	MSOffice	92.28	94.13	93.19	77.74	72.27	74.91
RePE (n=2)	PDF	99.03	99.59	99.31	98.98	97.57	98.27
	HWP	83.61	99.42	90.83	89.37	19.95	32.61
	MSOffice	93.07	96.70	94.85	80.29	65.15	71.94

Between the NoPE and RePE, when the *n* is fixed, the RePE exhibited better performance in all metrics. This indicates that the relative positional embeddings convey informative representations for malware detection. The NoPE worked better than any other positional encodings according to the work of Amirhossein Kazemnejad et al. [25], but it was a comparison between decoder-based LMs. As the encoder-based LM does not follow the auto-regressive objective, the relative positional embeddings play an important role allowing the LM to learn sequential and positional patterns from the byte sequences.

We also tried n∈{1,2} of n-grams as shown in Table 3, and found that the unigram (i.e., *n* = 1) gives better performance. The byte-level n-grams is essentially similar to the character-level n-grams in the NLP field. The character-level n-grams (or sub-character-level) have shown quite successful results for some languages (e.g., Chinese, Japanese) [30–32], where such languages have a common characteristic that they do not have a word boundary; they do not use white space between characters to separate words. Likewise, the sub-sequences of bytes are sampled without considering operation boundaries. The byte-level unigram seems to allow the model to avoid violating the operation boundaries and does not harm the semantic patterns within the sequence because the operation codes of programming languages are less complex than natural language sentences.

To summarize, we found that the best settings of the positional encoding and byte-level n-grams are the RePE and unigram tokenization. Besides this, the most fascinating finding is that the ByteEnc is pre-trained only with HWP sub-sequences but works well for malware detection with other different formats (e.g., PDF and MSOffice). It is consistent with the work of Young-Seob Jeong et al. [20] that utilized byte sequences of different file formats to improve malware detection performance. On the other hand, we observed worse performance for the ‘malware’ class in the HWP format compared to other formats. This may be due to the fact that HWP byte streams are significantly longer than those of other formats, and the imbalanced precision and recall result from the highly skewed distribution of HWP files, as shown in Table 1.

### Pre-training algorithms

**Fig 4:**
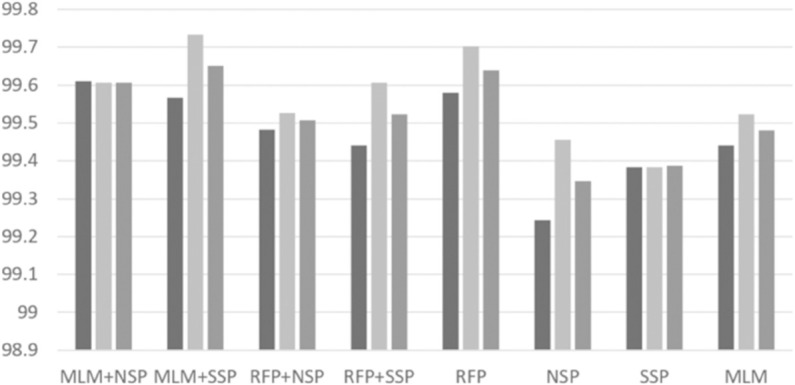
Averaged precision, recall, and F1 scores of ‘normal’ class on PDF format with different pre-training strategies, where the three boxes of each pre-training combination indicate precision, recall, and F1 scores, respectively.

**Fig 5:**
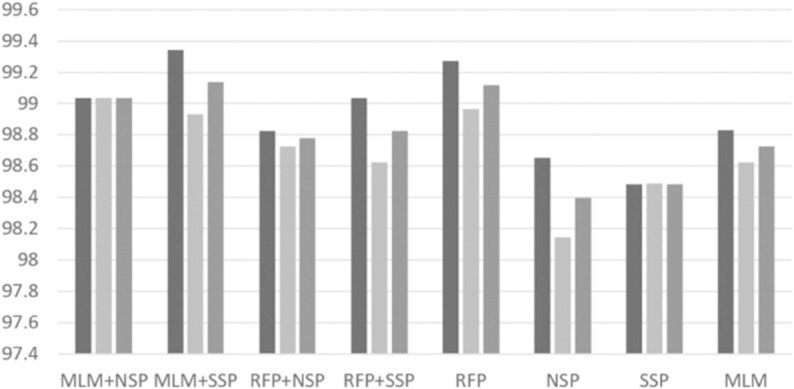
Averaged precision, recall, and F1 scores of ‘malware’ class on PDF format with different pre-training strategies, where the three boxes of each pre-training combination indicate precision, recall, and F1 scores, respectively.

**Fig 6:**
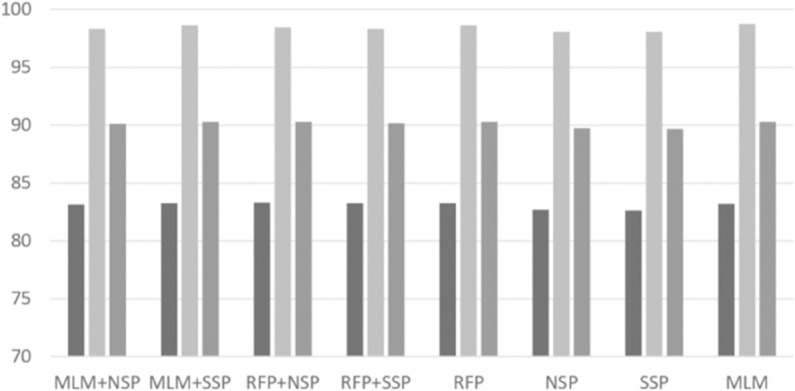
Averaged precision, recall, and F1 scores of ‘normal’ class on HWP format with different pre-training strategies, where the three boxes of each pre-training combination indicate precision, recall, and F1 scores, respectively.

**Fig 7:**
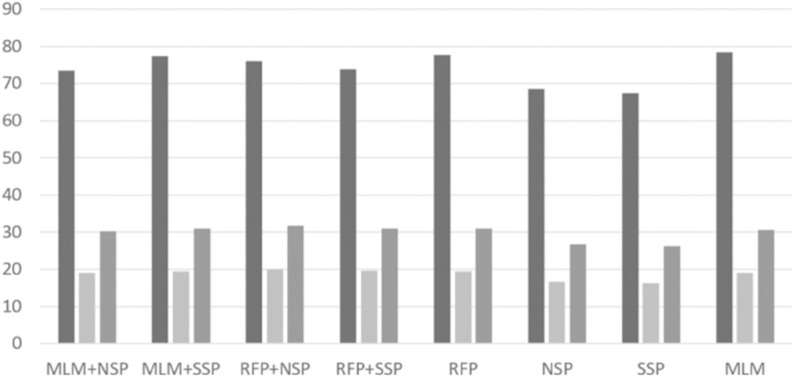
Averaged precision, recall, and F1 scores of ‘malware’ class on HWP format with different pre-training strategies, where the three boxes of each pre-training combination indicate precision, recall, and F1 scores, respectively.

**Fig 8:**
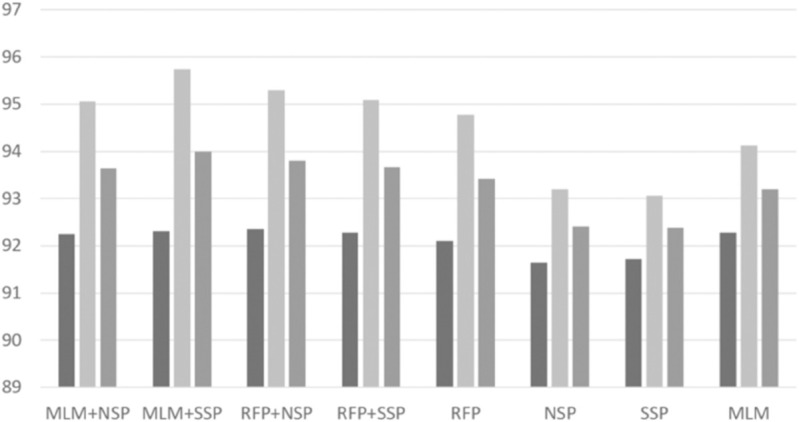
Averaged precision, recall, and F1 scores of ‘normal’ class on MSOffice format with different pre-training strategies, where the three boxes of each pre-training combination indicate precision, recall, and F1 scores, respectively.

**Fig 9:**
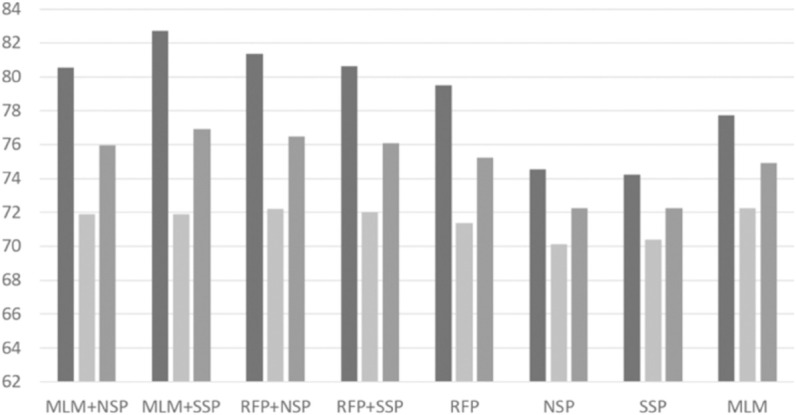
Averaged precision, recall, and F1 scores of ‘malware’ class on MSOffice format with different pre-training strategies, where the three boxes of each pre-training combination indicate precision, recall, and F1 scores, respectively.

With the best RePE and unigram tokenization settings, we performed intensive experiments to investigate possible combinations of pre-training algorithms. Based on a grid search, we set *p*_*rfp*1_ = 0.15 and *p*_*rfp*2_ = 0.5 for the RFP algorithm. The per-format performance of ‘normal’ and ‘malware’ classes are depicted in Figs 4, 5, 6, 7, 8, and 9. There are several key observations. First, among the token-level algorithms such as MLM and RFP, our proposed RFP generally shows superior performance; for example, for the ‘normal’ class in PDF format, the RFP algorithm showed approximately 0.15%p higher F1 score than the MLM algorithm. Likewise, among the sequence-level algorithms, our SSP outperforms the NSP algorithm. Second, compared to the token-level algorithms, the sequence-level algorithms (e.g., NSP and SSP) give the worst performance when they are adopted alone without combinations. This is reasonable as many previous studies found that the NSP algorithm may not contribute much to the down-stream performance. Third, the best combination is the MLM+SSP. This can be explained that the SSP algorithm makes the model to incorporate intra-sequence information so that the model is able to deal with malicious operations scattered over a sequence. This eventually allows the model to predict the original bytes more accurately in the MLM algorithm.

### Comparison to CNNs and a bigger language model

**Fig 10:**
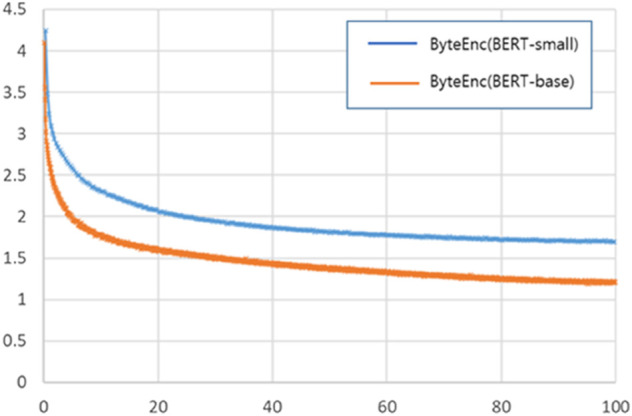
Loss curve during pre-training, where the x-axis represents the epoch and the y-axis represents the training loss.

Since previous models for malware detection using byte sequences have primarily been convolutional neural networks (CNNs), we compared our best model (i.e., ByteEnc pre-trained using MLM+SSP) with two CNN-based models: MalConv [10] and SPAP [21]. Both CNNs were trained using cross-entropy loss for 10 epochs, and their drop-out rate was 0.5, batch size was 10, and we followed default settings for other hyperparameters. Like ByteEnc, the input length for the CNNs was set to 512. The ByteEnc model used in our experiments was the size of BERT-small (29M parameters). Additionally, we tested a larger ByteEnc model with the same size as BERT-base (110M parameters) featuring 12 encoder layers. Table 4 summarizes the results of the CNNs and ByteEnc models of different sizes. Compared to the CNNs, the ByteEnc models consistently demonstrated significantly better performance across all file formats, as reflected in the Macro F1 scores. This result suggests that ByteEnc effectively transfers the knowledge of byte sequences learned during pre-training to the malware detection task. One potential concern is the relatively lower performance of the HWP format. However, as mentioned earlier, HWP files tend to have significantly longer sequences than other formats, making malware detection more challenging since malicious actions may be spread across these long sequences. In future work, we plan to extend our approach to support longer sequence lengths. Another noteworthy observation is that ByteEnc (BERT-base) generally outperforms ByteEnc (BERT-small). Although ByteEnc (BERT-base) shows slightly lower performance in the PDF format, it achieves greater performance gains in the other two formats. Notably, both ByteEnc (BERT-base) and ByteEnc (BERT-small) were pre-trained on the same dataset, suggesting that a larger language model is better at learning and capturing the sequential patterns underlying byte sequences, ultimately leading to improved performance. This is also supported by Fig 10, which shows that the training loss of ByteEnc (BERT-base) has decreased more than that of ByteEnc (BERT-small).

**Table 4:** Malware detection performance of convolutional neural networks (CNN) and ByteEnc (our method).

Model	Format	Macro F1	Normal			Malware		
			Precision	Recall	F1	Precision	Recall	F1
SPAP [21]	PDF	98.16	99.03	98.82	98.93	97.13	97.63	97.38
	HWP	56.07	92.32	53.67	67.87	30.33	81.87	44.26
	MSOffice	79.23	92.25	87.97	90.06	63.59	73.97	68.39
MalConv [10]	PDF	96.71	96.92	99.41	98.15	98.46	92.26	95.26
	HWP	57.10	90.84	57.47	70.40	30.70	76.46	43.80
	MSOffice	75.20	89.87	86.99	88.41	58.84	65.48	61.98
ByteEnc (BERT-small)	PDF	**99.40**	99.57	99.73	99.65	99.34	98.93	99.14
	HWP	60.62	83.23	98.59	90.26	77.28	19.37	30.97
	MSOffice	85.47	92.31	95.74	93.99	82.73	71.91	76.94
ByteEnc (BERT-base)	PDF	99.29	99.52	99.65	99.59	99.14	98.83	98.98
	HWP	**61.07**	83.31	99.21	90.57	85.87	19.33	31.56
	MSOffice	**86.34**	92.40	96.57	94.44	85.65	72.02	78.24

## Conclusions

In this paper, we introduced a new language model that is pre-trained with only byte sequences, thereby protecting Web users from the threat of malicious attacks. The language model, ByteEnc, is pre-trained with the byte sequences of HWP documents, but we demonstrated that it worked well for malware detection for other different formats such as PDF and MS Office. We also designed new pre-training algorithms and investigated combinations of the pre-training algorithms. We conducted extensive experiments and demonstrated that ByteEnc significantly outperformed previous convolutional neural networks (CNNs) with a large performance gap. We believe our language model will serve as a foundation model for malware detection services for non-executables.

Although we experimented with ByteEnc in two sizes (e.g., BERT-base and BERT-small), there is still ample room for performance improvement with much larger models. In this paper, we set the input length to 512, which may not be sufficient to capture malicious actions across the byte sequences, potentially resulting in overall performance degradation in practical applications. In particular, despite high precision for the normal class, there remains a risk that its deployment in real-world security services could lead to significant financial and societal costs because of the low reliable performance (i.e., low sensitivity). We believe that the fundamental solution for this limitation is to keep collecting more balanced dataset, so that the models learn more about the malicious cases.

In future work, we plan to extend our research to develop a more powerful encoder-based model that can process longer byte sequences. Furthermore, we will continue collecting and annotating balanced dataset, and we expect that pre-training on larger datasets with more diverse formats will make a significant contribution to advancements in the field of malware detection.
